# High Dose Cyclophosphamide Treatment for Autoimmune Disorders

**DOI:** 10.1100/tsw.2002.863

**Published:** 2002-06-28

**Authors:** Robert A. Brodsky

**Affiliations:** The Sidney Kimmel Comprehensive Cancer Center at Johns Hopkins, Division of Hematologic Malignancies, Baltimore, MD 21231-1000, USA

**Keywords:** high dose cyclophosphamide, autoimmune disease, aplastic anemia, autoimmune hemolytic anemia, bone marrow transplantation

## Abstract

High-dose cyclophosphamide (200 mg/kg) was initially developed as a conditioning regimen for allogeneic bone marrow transplantation. Recently, high-dose cyclophosphamide without bone marrow transplantation has been employed as a method to induce durable treatment-free remissions in severe aplastic anemia and a variety of other severe autoimmune disorders. The premise underlying this approach is that high-dose cyclophosphamide is maximally immunosuppressive, but not myeloablative. Early hematopoietic stem cells are spared the cytotoxicity of cyclophosphamide because of their high levels of aldehyde dehydrogenase, an enzyme that confers resistance to the drug. Conversely, autoimmune effector cells (T cells, B cells, and NK cells) are exquisitely sensitive to high-dose cyclophosphamide because of their relatively low levels of aldehyde dehydrogenase. Intensive investigation is underway to determine which autoimmune disorders will most benefit and where in the natural history of these diseases to employ this rapidly developing therapy.

## INTRODUCTION

Autoimmune diseases afflict more than 8 million people in the U.S. and impact on virtually every medical specialty. While many autoimmune disorders (e.g., vitiligo, thyroiditis, pernicious anemia)

are relatively indolent and easily managed, severe cases of systemic lupus erythematosus, rheumatoid arthritis, aplastic anemia, multiple sclerosis, etc., can cause severe morbidity and even mortality. As eradication of severe autoimmune diseases by conventional immunosuppressive therapy is unusual, most of these patients must endure lifelong immunosuppressive therapy with its attendant complications. Thus, novel treatments for severe autoimmune diseases that reduce disease activity and avoid lifelong immunosuppression are needed.

The ability of allogeneic bone marrow transplantation (performed principally for a life-threatening hematopoietic disease) to eradicate a concurrent autoimmune disease[1,2], coupled with the demonstration that syngeneic bone marrow transplantation in autoimmune animal models can eradicate autoimmunity, has stimulated interest in applying bone marrow transplantation for the treatment of autoimmune diseases[3,4,5]. In bone marrow transplantation, the source of hematopoietic stem cells may be from a normal donor (allogeneic) or from the patient (autologous). Autologous stem cell transplantation is more widely applicable because it does not require an HLA-matched donor and it is associated with less morbidity and mortality because it avoids the risk of graft-vs.-host-disease. Thus, most centers are studying autologous, rather than allogeneic, stem cell transplantation for treating autoimmune diseases. The three major components to autologous stem cell transplantation are as follows: (1) stem cell mobilization and collection, (2) “conditioning” with high-doses of chemotherapy in order to eradiate autoreactive effector cells or tumor cells, and (3) stem cell infusion. Mobilization refers to the administration of chemotherapy or hematopoietic growth factors in order to collect peripheral blood progenitor cells for transplantation. However, for treating autoimmunity with autologous stem cell transplantation, the therapeutic efficacy (high-dose immunosuppression/immunoablation) is derived entirely from the conditioning regimen (high-dose chemotherapy); hematopoietic stem cells are used principally as a rescue procedure to avoid prolonged periods of aplasia. A major concern with autologous stem cell transplantation for autoimmunity is that the mobilized product contains a large number of effector cells (lymphocytes) which may in theory re-establish the disease[6,7,8]. Therefore, many groups are investigating strategies to purge the autograft of contaminating lymphocytes. Due to its potent immunosuppressive activity, high-dose cyclophosphamide (50 mg/kg/day $\times$ 4 days) is the foundation of most conditioning regimens used for autoimmunity[9]; in many instances it is the sole cytotoxic agent employed[9,10,11]. High-dose cyclophosphamide is nonmyeloablative; thus, it can be used without stem cell rescue and circumvent the risk of reinfusing autoreactive lymphocytes with the autologous graft. This mini-review article will communicate the scientific and clinical rationale for using high-dose cyclophosphamide without stem cell transplantation to treat autoimmune disease.

## PHARMACOLOGY OF HIGH-DOSE CYCLOPHOSPHAMIDE

The unique pharmacology of high-dose cyclophosphamide accounts for its potent immunosuppressive properties and its ability to spare hematopoietic stem cells. High-dose cyclophosphamide was originally developed as conditioning for allogeneic bone marrow transplantation because of its ability to break tolerance and facilitate engraftment[12]. As a prodrug, cyclophosphamide is converted to 4-hydroxycyclophosphamide and its tautomer aldophosphamide in the liver (Fig. 1). These compounds freely diffuse into the cell and are converted to the active compound phosphoramide mustard, or they are inactivated by aldehyde dehydrogenase to form the inert carboxyphosphamide. Lymphoid cells, including natural killer cells, B and T lymphocytes, have low levels of aldehyde dehydrogenase and are rapidly killed by high doses of cyclophosphamide. However, primitive hematopoietic stem cells possess high levels of aldehyde dehydrogenase rendering them highly resistant to cyclophosphamide[13,14,15]. Therefore, high-dose cyclophosphamide is highly immunosuppressive, but not myeloablative; endogenous hematopoietic stem cells will reconstitute hematopoiesis without the need for a stem cell graft.

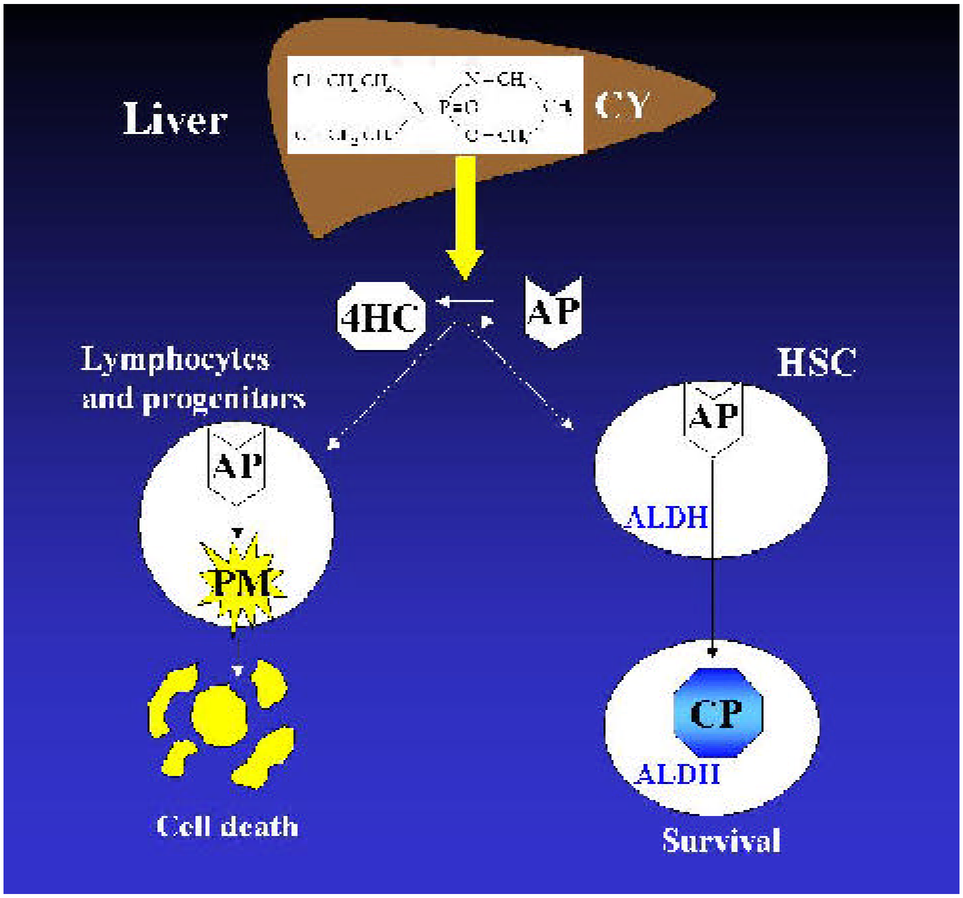

**FIGURE 1.** Pharmacology of high-dose cyclophosphamide. Cyclophosphamide (CY) is converted in the liver to 4 hydrodroxycyclophosphamide (4HC) and its tautomer aldophosphamide (AP), which diffuses into cells. Hematopoietic stem cells (HSC) have high-levels of aldehyde dehydrogenase (ALDH) which converts AP into the inert compound carboxyphosphamide (CP). Lymphocytes and progenitor cells have low levels of ALDH. In these cells AP is converted into the active compound phosphoramide mustard (PM) leading to cell death.

## HIGH-DOSE CYCLOPHOSPHAMIDE FOR SEVERE APLASTIC ANEMIA

Aplastic anemia is a life-threatening bone marrow failure disorder. Although not originally considered an autoimmune disease, ample clinical and laboratory data support the hypothesis that in most cases of aplastic anemia autoreactive cytotoxic T lymphocytes are directly responsible for the bone marrow destruction[16,17,18,19,20,21]. Aplastic anemia is classified as severe (SAA) when the bone marrow cellularity is <25% and there is marked peripheral cytopenias in at least two hematopoietic lineages (neutrophil count <0.5 $\times$ $10^9$/l, platelet count <20 $\times$ $10^9$/l and absolute reticulocyte count of <60,000)[22]. With supportive care alone, most patients with SAA die within a year of diagnosis; consequently, prompt therapeutic intervention is necessary. Based on the pathophysiology of aplastic anemia (autoimmune destruction of hematopoietic precursors) three major therapeutic approaches have emerged: (1) high-dose cyclophosphamide (50 mg/kg/day $\times$ 4 days) followed by allogeneic bone marrow transplantation, (2) immunosuppressive therapy (usually consisting of antithymocyte globulin and cyclosporine), and (3) high-dose cyclophosphamide without bone marrow transplantation.

Bone marrow transplantation was the first therapy to achieve a high probability of durable treatment-free remissions in SAA[23]. By the mid-1970s, conditioning with high-dose cyclophosphamide followed by allogeneic bone marrow transplantation became the treatment of choice for young patients with SAA. Shortly thereafter, reports of autologous hematopoietic reconstitution following allogeneic bone marrow transplantation for SAA appeared, suggesting that high-dose cyclophosphamide alone could restore normal hematopoiesis in aplastic anemia[24,25,26]. In 1976, a case report in the *New England Journal of Medicine* described a patient with SAA who was successfully treated with high-dose cyclophosphamide alone[27]. Despite the apparent success of high-dose cyclophosphamide in treating SAA, the approach received little attention for another two decades due, in part, to the improving results with allogeneic BMT and the emergence of antithymocyte globulin and cyclosporine as a treatment for SAA. In children and young adults, allogeneic BMT from an HLA-matched sibling cures 75–90% of SAA patients; however, in SAA patients older than 40 years, the cure rate following an HLA-matched sibling transplant decreases to approximately 50%, due to mainly to the increased risk of graft-vs.-host-disease. Ultimately, the high rate of relapse and late clonal diseases (myelodysplastic syndromes and paroxysmal nocturnal hemoglobinuria) associated with immunosuppressive therapy and the high risk of mortality and severe graft-vs.-host-disease associated with unrelated and mismatched bone marrow transplantation led to a renewed interest in high-dose cyclophosphamide.

The first reported clinical trial of high-dose cyclophosphamide without bone marrow transplantation for the treatment of SAA was initiated in the late 1970s during a time period when antithymocyte globulin was temporarily unavailable in the U.S. The results, published in 1996, demonstrated that high-dose cyclophosphamide produced durable complete remissions in seven of the ten SAA patients; none of these patients had received prior immunosuppressive therapy[28]. With a median follow-up exceeding 10 years, no patient in the trial had relapsed, had required additional therapy, or had acquired a secondary clonal disease demonstrating that high-dose cyclophosphamide has the potential to cure SAA. The trial's small size and the fact that the patients were not treated consecutively evoked concern that the patients were highly selected and/or that these data would not apply to the majority of patients with SAA. A few years later, Li et al. reported on the use of high-dose cyclophosphamide in two adult patients from China with SAA[29]. The first patient was previously untreated and achieved a complete remission; the second patient, who was refractory to immunosuppressive therapy died of an intracerebral hemorrhage. In Mexico, Jaime-Perez et al. employed high-dose cyclophosphamide therapy in five children with SAA who were previously unresponsive to androgen therapy (four patients) or cyclosporine therapy (one patient)[30]. A complete remission was achieved in three of the five children with follow-up of 23, 24, and 40 months, respectively. Two patients died; one on day 8 of mucormycosis and the other at 3 months from a cerebral hemorrhage. There were no relapses or late clonal diseases in these patients.

The initial enthusiasm for high-dose cyclophosphamide in treating SAA was tempered after a study at The National Institutes of Health (NIH) reported a high incidence of invasive fungal infections when high-dose cyclophosphamide was combined with 6 months of oral cyclosporine. These investigators initiated a randomized study of high-dose cyclophosphamide/cyclosporine (CSA) vs. antithymocyte globulin (ATG)/CSA with a statistical goal of enrolling 91 patients to each arm[31]. The study was aborted after enrolling 31 total patients due to greater toxicity (more infections and longer duration of aplasia) in the cyclophosphamide/cyclosporine arm; however, no stopping rules, primary endpoints or secondary endpoints were reached. Only 13 patients in the cyclophosphamide/CSA arm and 12 patients in the ATG/CSA arm were evaluable at 6 months. The 6-month mortality in the cyclophosphamide/CSA arm (3/13) was not significantly different from the mortality in the ATG/CSA arm (1/12), ($p$ = 0.3%). There were five relapses and one case of MDS in the ATG/CSA arm, and one relapse in the cyclophosphamide/CSA arm. The study demonstrated slower hematopoietic recovery and a greater need for blood products and antibiotics in the cyclophosphamide/CSA arm, but it was not sufficiently powered to address differences in response rate, response duration, overall survival, and evolution to secondary clonal disease. The concurrent use of CSA with cyclophosphamide may have increased toxicity[32]. Moreover, because CSA blocks the induction of tolerance, a potential mechanism of action for cyclophosphamide, it probably should not be added to the high-dose cyclophosphamide regimen when treating aplastic anemia[33].

More recently, investigators at Johns Hopkins University, MCP Hahnemann University, and The University of Maryland reported the results of the largest trial to date studying the efficacy of high-dose cyclophosphamide for the treatment of SAA[34]. A total of 19 patients with a median age of 47 (range, 18–68) years were enrolled. The survival probability was 84% at 24 and 48 months, with a median follow-up of 34 months in surviving patients. The probability of transfusion independence was 73% at 24 months and the probability of complete response was 65% at 50 months. There were three deaths; two from presumed fungal infections on days 26 and 57 after cyclophosphamide, and one from an intracerebral hemorrhage on day 67 after cyclophosphamide. All three of these patients were greater than 50 years of age and had active infections at the time of treatment. No other patients developed invasive fungal infections. All responding patients remain in a treatment-free remission with Karnofsky scores of 100. Hematopoietic reconstitution in these patients was gradual, taking a median of 49 days after treatment to achieve $0.5 \times 10^9$ neutrophils/l and median 36 months to achieve a complete remission. The median time to transfusion independence of red cells and platelets was 11 months. Of the 13 patients with greater than 1 year follow-up, 12 are in treatment-free remissions (9 complete responses and 3 partial responses). No patient has relapsed, required additional immunosuppressive therapy, developed paroxysmal nocturnal hemoglobinuria, myelodysplasia, or other malignancies. We have also successfully used high-dose cyclophosphamide to salvage SAA patients who relapsed or were unresponsive to ATG/CSA; however, the response rate to high-dose cyclophosphamide is best when it is used as front-line therapy (unpublished data). Thus, while the early toxicity of high-dose cyclophosphamide may exceed that of ATG/CSA, the quality and duration of response in uncontrolled trials appears to be superior. Further study of high-dose cyclophosphamide in treating SAA is necessary before accepting this approach as standard therapy.

## HIGH-DOSE CYCLOPHOSPHAMIDE FOR OTHER AUTOIMMUNE DISEASES

The ability of high-dose cyclophosphamide to induce durable complete remissions in one autoimmune disease (aplastic anemia) has broad implications for the management of other autoimmune and possibly alloimmune conditions. Many centers world-wide are using high-dose cyclophosphamide followed by peripheral blood stem cell transplantation to treat a variety of refractory severe autoimmune diseases[8,9,10]. A major concern with this approach is that autoreactive effector cells that are reinfused with the autograft may lead to relapse; thus, many investigators are exploring methods to purge the autograft of lymphocytes[7]. Peripheral blood stem cells are essential as a rescue procedure with myeloablative conditioning regimens, such as, cyclophosphamide/total body irradiation or busulfan/cyclophosphamide. However, stem cell transplantation conditioning regimens for treating autoimmune disorders are predominantly nonmyeloablative; high-dose cyclophosphamide alone or high-dose cyclophosphamide combined with antithymocyte globulin are the most frequently employed regimens.

To test this approach, we treated eight patients with high-dose cyclophosphamide for a variety of severe refractory autoimmune disorders (two systemic lupus erythematosus, two Felty syndrome, one immune thrombocytopenia, two autoimmune hemolytic anemia, and one chronic inflammatory demyelinating polyneuropathy)[35]. Seven patients showed marked clinical improvement: five achieved a complete remission and two achieved a partial remission. Hematopoietic reconstitution was rapid. The median time to a neutrophil count of 500/µl and platelet transfusion independence was 17 and 16 days after the last dose of cyclophosphamide, respectively. High-dose cyclophosphamide without stem cell transplantation was next reported to induce durable complete remissions in patients with paraneoplastic pemphigus[36] and pemphigus vulgaris[37]. Following high-dose cyclophosphamide, the patient with paraneoplastic pemphigus did not require blood products and recovered to a neutrophil count of greater than 500/µl by day 15 after high-dose cyclophosphamide. The patient with pemphigus vulgaris began to recover neutrophils by day 9; he received two platelet transfusions, but did not require red cell transfusions. In both patients, the pathogenic autoantibodies specific for the disease became undetectable after high-dose cyclophosphamide. Durable remission following high-dose cyclophosphamide has also been reported in refractory autoimmune hemolytic anemia[38]. Moyo et al. treated nine patients with high-dose cyclophosphamide for refractory autoimmune hemolytic anemia; seven had an IgG warm autoantibody, one had an IgM cold agglutinin, and one had both warm and cold agglutinin disease. The median hemoglobin at the time of treatment was 6.7 (range; 5–10) g/dl and eight of the nine patients were dependent on erythrocyte transfusions. The median times to a neutrophil count of 500/µl and to platelet transfusion independence after high-dose cyclophosphamide was 16 and 15 days, respectively. All patients responded and became transfusion independent; six patients achieved a complete remission (normal untransfused hemoglobin for age and sex) and three patients achieved a partial remission (hemoglobin >10.0 g/dl without support of transfusions). There were no relapses at a median follow-up of 15 (range; 4–29) months and seven of the nine patients were able to discontinue steroids.

Brannagan and colleagues treated four patients with high-dose cyclophosphamide for refractory chronic inflammatory polyneuropathy; three had progressive disease and one had relapsing disease[39]. All four patients had improvement of strength and functional status, with three of four showing improvement in summated compound motor action potential. All patients were able to discontinue other immunomodulatory medications.

High-dose cyclophosphamide may also have the potential to eradicate alloimmunization, a major problem in patients who require chronic blood transfusions and in patients being considered for organ transplantation[40]. Five patients with SAA who were refractory to platelet transfusions due to HLA-specific antibodies were studied before and after treatment with high-dose cyclophosphamide. Complete remission of the SAA was achieved in four of these five patients. All four responders demonstrated a marked reduction in anti-HLA antibody titer after high-dose cyclophosphamide; in three of these patients the antibody was completely eradicated suggesting that high-dose cyclophosphamide may have the potential to treat alloimmune conditions.

## CONCLUSION

High-dose cyclophosphamide is a potent, nonmyeloablative, immunosuppressive regimen that leads to durable treatment-free remissions in the majority of patients with untreated SAA. Durable complete remissions after high-dose cyclophosphamide have also been observed in a variety of other autoimmune and even alloimmune conditions. A major advantage of this approach over peripheral blood stem cell transplantation is that it eliminates the risk of reinfusing autoreactive effector cells with the autograft without significantly prolonging the duration of aplasia. The duration of aplasia following high-dose cyclophosphamide in autoimmune diseases other than SAA is brief, usually 10–14 days, and is extremely well tolerated. Nevertheless, more experience and longer follow-up are necessary in individual autoimmune disease such as systemic lupus erythematosus, pemphigus, and autoimmune hemolytic anemia etc., in order to determine where in the natural history of these disorders to employ high-dose cyclophosphamide. A phase III randomized controlled trial of high-dose cyclophosphamide vs. monthly pulse dose cyclophosphamide for moderate to severe lupus is currently underway.

## BIOSKETCH

**Robert A. Brodsky** is Associate Professor of Oncology and Medicine, The Sidney Kimmel Comprehensive Cancer Center at Johns Hopkins. Dr. Brodsky received his M.D. degree from Hahnemann University and completed his Internal Medicine residency at Vanderbilt University. He went on to do his Hematology Fellowship at the National Institutes of Health and an Oncology Fellowship at Johns Hopkins. Dr. Brodsky's laboratory and clinical research focuses on the mechanisms of bone marrow failure and the treatment of these disorders. He has pioneered the use of high-dose cyclophosphamide to treat severe aplastic anemia and other autoimmune diseases.
